# A framework for the investigation of pleiotropy in two‐sample summary data Mendelian randomization

**DOI:** 10.1002/sim.7221

**Published:** 2017-01-23

**Authors:** Jack Bowden, Fabiola Del Greco M, Cosetta Minelli, George Davey Smith, Nuala Sheehan, John Thompson

**Affiliations:** ^1^ MRC Integrative Epidemiology UnitUniversity of BristolU.K.; ^2^ Center for BiomedicineEURAC researchBolzanoItaly; ^3^ Population Health and Occupational DiseaseNHLI, Imperial CollegeLondonU.K.; ^4^ Department of Health SciencesUniversity of LeicesterLeicesterU.K.

**Keywords:** instrumental variables, Mendelian randomization, meta‐analysis, MR‐Egger regression, pleiotropy

## Abstract

Mendelian randomization (MR) uses genetic data to probe questions of causality in epidemiological research, by invoking the Instrumental Variable (IV) assumptions. In recent years, it has become commonplace to attempt MR analyses by synthesising summary data estimates of genetic association gleaned from large and independent study populations. This is referred to as two‐sample summary data MR. Unfortunately, due to the sheer number of variants that can be easily included into summary data MR analyses, it is increasingly likely that some do not meet the IV assumptions due to pleiotropy. There is a pressing need to develop methods that can both detect and correct for pleiotropy, in order to preserve the validity of the MR approach in this context. In this paper, we aim to clarify how established methods of meta‐regression and random effects modelling from mainstream meta‐analysis are being adapted to perform this task. Specifically, we focus on two contrastin g approaches: the Inverse Variance Weighted (IVW) method which assumes in its simplest form that all genetic variants are valid IVs, and the method of MR‐Egger regression that allows all variants to violate the IV assumptions, albeit in a specific way. We investigate the ability of two popular random effects models to provide robustness to pleiotropy under the IVW approach, and propose statistics to quantify the relative goodness‐of‐fit of the IVW approach over MR‐Egger regression. © 2017 The Authors. *Statistics in Medicine* Published by JohnWiley & Sons Ltd

## 1 Introduction

**Figure 1:**
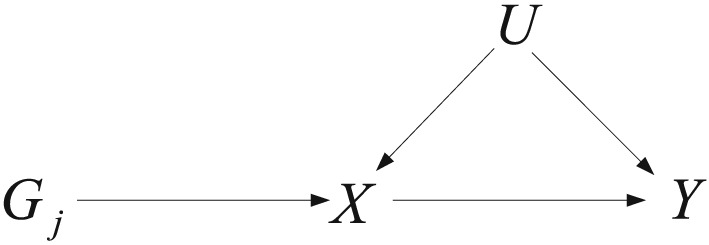
Illustrative diagram showing the hypothesised causal relationship between a genetic variable *G*_*j*_, environmental exposure *X* and outcome *Y*.

The fundamental aim of Epidemiology is to determine the root causes of illness, with the focus of many epidemiological analyses being to examine whether an environmental exposure modifies the severity, or the risk of, disease. Of course, it is well known that causal conclusions cannot strictly be drawn from mere statistical associations between an exposure and outcome, unless all possible confounders of the association are identified, perfectly measured and appropriately adjusted for. Mendelian randomization (MR) 1, 2 offers an alternative way to probe the issue of causality in epidemiological research, by using additional genetic information satisfying the Instrumental Variable (IV) assumptions. Briefly, the genetic data must both predict the exposure and predict the outcome only through the exposure. Figure 1 shows an illustrative causal diagram relating a single nucleotide polymorphism (SNP) *G*_*j*_ to an exposure, *X* and outcome, *Y*, in the presence of an unmeasured confounder, *U*. Here, a single *U* is used to denote the combined influence of all unmeasured confounders.

To state the IV assumptions more formally: the *j*th SNP must be: associated with *X* (IV1); independent of *U* (IV2) and independent of *Y* given *X* and *U*(IV3). These assumptions are encoded by the arrows in Figure 1. If IV1–IV3 are satisfied for variant *G*_*j*_, then traditional IV methods can be employed to reliably test for a causal effect using *G*_*j*_,*X* and *Y* alone, without any attempt to adjust for *U* at all.

An array of sophisticated techniques exist for estimating the causal effect with individual participant data. However, the sharing of such data is often impractical and in recent years, it has become much more common to attempt MR analyses using summary data estimates of SNP‐exposure and SNP‐outcome associations based on large and independent populations 3, 4. This is referred to as two‐sample summary data MR 5, 6. The SNPs in question are usually those identified as ‘hits’ in separate genome wide association studies (GWAS). They are generally picked from distinct genomic regions in order to be mutually independent, or not in linkage disequilibrium (LD). The veracity of this assumption can be further scrutinised by using external data resources that catalogue LD structure across the genome (see for example http://www.internationalgenome.org/). In many cases, this restriction still allows hundreds of SNPs to be included in the analysis. If all of the incorporated genetic variables provide independent and unbiased estimates of the same causal effect, then summary data MR can be reliably reduced to a fixed effect meta‐analysis of the set of causal estimates obtained using each genetic variant in turn. Many assumptions are necessary for this to be true, however. For example, the SNPs must at the very least be uncorrelated and valid IVs. They must also collectively satisfy additional modelling restrictions with respect to the exposure and outcome, which will be discussed in detail in Section 2. Unfortunately, due to the sheer number of variants that can now be easily included in such MR analyses (often with limited knowledge of their functional role), it is increasingly likely that some fail to meet the fundamental assumption of being a valid IV, due to pleiotropy.

**Figure 2:**
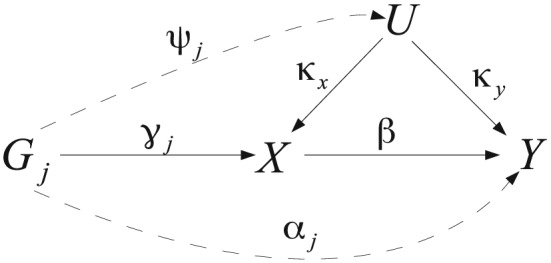
Illustrative diagram showing the causal and parametric relationships between genetic variable *G*_*j*_, *X* and *Y*. Solid lines on their own define *G*_*j*_ as a valid IV, but the addition of dashed lines indicate violations of the IV assumptions.

Pleiotropy occurs when a single SNP affects multiple phenotypes related to the outcome 2, 7, and is a potential barrier to obtaining reliable inferences via MR. Crucially, however, only pleiotropy of a certain type poses a threat. For example, in Figure 1 *G*_*j*_ is a valid IV, but when *X* causally affects *Y*,*G*_*j*_ will be associated with both *X* and *Y*(through *X*). Indeed, MR exploits this very fact to test for causality. Pleiotropy *is* problematic when a SNP affects *Y* through phenotypic pathways other than *X*, because this could invalidate its use as an IV. This is illustrated by the addition of dashed lines in Figure 2. For example the dashed line linking variant *G*_*j*_ to *U* would make *G*_*j*_ invalid due to violation of IV2, and the dashed line linking variant *G*_*j*_ to *Y* would make *G*_*j*_ invalid to due violation of IV3. From now on, we use the term ‘pleiotropy’ to refer to IV violations of this sort.

There is a pressing need to develop methods that can both detect and correct for pleiotropy in order to preserve the validity of the MR approach. Indeed, some progress has already been made towards this goal 8, 9, 10, 11. The aim of this paper is to clarify how established methods of meta‐regression and random effects modelling from mainstream meta‐analysis have been adapted to perform this task. Specifically, we focus on two contrasting approaches: The Inverse Variance Weighted (IVW) method 6, 12 which assumes in its most basic form that IV1–3 hold (e.g. no pleiotropy) and is equivalent to a fixed effect meta‐analysis; and secondly a simple adaptation to the MR context of the method of Egger regression 13 used to adjust for small study bias in meta‐analysis (referred to as MR‐Egger 9. In doing so, we attempt to make use of, extend and connect the work of Rücker et al. 14 and Henmi and Copas 15 for probing the issue of small study bias in meta‐analysis with the work of Del Greco et al 16 and Bowden et al 9, 17 for detecting and accounting for pleiotropy in summary data MR analysis.

In Section 2, we clarify the assumptions necessary for a two‐sample summary data MR analysis, by starting from an underlying model for data at the individual level. In Sections 3 and 4, we explore how the IVW and MR‐Egger methods can be applied to pleiotropy‐affected summary data, by drawing on the meta‐analytical framework of Rücker et al 14. In Section 5, we look at the issue of estimating the causal effect in practice, when some of the crucial assumptions are violated. In Section 6, we illustrate the methods discussed using an application that probes the causal effect of plasma urate levels on cardiovascular disease risk. We conclude with a discussion and point to future research.

## 2 Modelling assumptions

We start by writing the assumed data generating model for subject *i* out of *N*, linking genetic data on *L* uncorrelated variants *G*_*i*1_,…,*G*_*iL*_ ≡ *G*_*i*._, to their exposure *X*_*i*_ and outcome *Y*_*i*_ in the presence of an unobserved summary confounder *U*_*i*_, as represented in Figure 2. *X*,*Y* and *U* are assumed to be continuous and *G*_*ij*_ represents the number of alleles of SNP *j* assigned to subject *i*(0,1 or 2) at the point of conception. For simplicity, we ignore intercept terms in the models below:

(1) $$U_{i}|G_{i.}=\overset{L}{\underset{j=1}{\sum }}\psi_{j}G_{ij}+\varepsilon_{ui}$$

(2) $$X_{i}|U_{i},G_{i.}=\overset{L}{\underset{j=1}{\sum }}\gamma_{j}G_{ij}+\kappa_{x}U_{i}+\varepsilon_{xi}$$

(3) $$Y_{i}|X_{i},U_{i},G_{i.}=\beta X_{i}+\kappa_{y}U_{i}+\overset{L}{\underset{j=1}{\sum }}\alpha_{j}G_{ij}+\varepsilon_{yi}$$

Here, *β* represents the causal effect of *X* on *Y* that we wish to estimate and *γ*_*j*_ represents the underlying true association between SNP *j* and the exposure *X* (i.e. not through *U*). We assume that the genetic data has been coded to reflect the number of exposure increasing alleles (be they ‘minor’ or 'major'), so that $\gamma_{j}⩾0$. We define IV1 to be satisfied for variant *j* when *γ*_*j*_ is non‐zero. Conversely, when they are non‐zero, the parameters *ψ*_*j*_ and *α*_*j*_ summarise all violations of IV2 and IV3 for SNP *j*, respectively. *κ*_*x*_ and *κ*_*y*_ represent the influence of all confounders on the exposure and outcome, respectively. Only when *κ*_*x*_ and *κ*_*y*_ are *both* non‐zero can confounding take effect, thus biasing a traditional observational analysis so that the association between *X* and *Y* is not equal in general to *β*(hence the need for IV methods). In the main body of this paper, we will interpret all parameters indexing these (and subsequent) models as fixed values in the ‘classical’ sense. Alternative Bayesian formulations are of course possible, a topic we return to in the discussion.

The focus of this paper is to consider the case where only summary data estimates of the SNP‐exposure and SNP‐outcome associations and their standard errors are available for each of the *L* variants. In order to derive models for these two sets of *L* summary data estimates, we firstly define *G*_ − *j*_ as the (*L* − 1)‐length vector of genetic variables without *G*_*j*_. We now re‐write models (2) and (3) as models for *X*|*G*_*j*_ and *Y*|*G*_*j*_ only, by marginalising over (*U*,*G*_ − *j*_) and (*X*,*U*,*G*_ − *j*_), respectively:

(4) $$\begin{array}{ccc} X_{i}|G_{ij} & =(\gamma_{j}+\kappa_{x}\psi_{j})G_{ij}+\varepsilon_{xij}^{\prime } & \\ \text{where}\;\varepsilon_{xij}^{\prime } & =\underset{l\neq j}{\sum }\left(\gamma_{l}+\kappa_{x}\psi_{il}\right)G_{il}+\kappa_{x}\varepsilon_{ui}+\varepsilon_{xi} \end{array}$$

(5) $$\begin{array}{ccc} Y_{i}|G_{ij} & =\left\{\beta (\gamma_{j}+\kappa_{x}\psi_{j})+\kappa_{y}\psi_{j}+\alpha_{j}\right\}G_{ij}+\varepsilon_{yij}^{\prime } & \\ \text{where}\;\varepsilon_{yij}^{\prime } & =\beta \varepsilon_{xij}^{\prime }+\underset{l\neq j}{\sum }\left(\kappa_{y}\psi_{l}+\alpha_{l}\right)G_{il}+\kappa_{y}\varepsilon_{ui}+\varepsilon_{yi} \end{array}$$

Now the coefficients of *G*_*ij*_ in (4) and (5) represent the underlying quantities that would be estimated in principle with summary data for SNP *j* under our assumed model. Note that all other terms have been subsumed into new residual error terms $\varepsilon_{xij}^{\prime }$ and $\varepsilon_{yij}^{\prime }$.

### 2.1 Two‐sample Mendelian randomization

It is becoming increasingly common to perform summary data MR analyses using sets of genetic association estimates with the exposure and outcome that have been gleaned from separate samples. The most natural way of justifying this approach is to assume that the SNP‐exposure and SNP‐outcome samples, whilst being independent, are homogeneous in the sense that models (4) and (5) hold for both. We will refer to them as sample ‘1’ and ‘2’, with *N*_1_ and *N*_2_ subjects, respectively. For clarity, we now write explicit models for the two sets of *L* SNP‐exposure and SNP‐outcome association estimates from the two samples. We refer to the *j*th SNP‐exposure association estimate as ${\hat{\gamma }}_{j}$(with variance $\sigma_{Xj}^{2}$) and the *j*th SNP‐outcome association estimate as ${\hat{\Gamma }}_{j}$(with variance $\sigma_{Yj}^{2}$):

(6) $$\text{Sample 1:}\;{\hat{\gamma }}_{j}=\gamma_{j}+k_{x}\psi_{j}+\varepsilon_{Xj},\;\mathrm{var}(\varepsilon_{Xj})=\sigma_{Xj}^{2}$$

(7) $$\text{Sample 2:}\;{\hat{\Gamma }}_{j}=\alpha_{j}+k_{y}\psi_{j}+\beta (\gamma_{j}+k_{x}\psi_{j})+\varepsilon_{Yj}\;\mathrm{var}(\varepsilon_{Yj})=\sigma_{Yj}^{2}$$

From (4) and (5), the magnitude of $\sigma_{Xj}^{2}$ and $\sigma_{Yj}^{2}$ are affected by the allele frequencies of SNPs in *G*_ − *j*_ and the sample size (*N*_1_ or *N*_2_). Because they relate to homogeneous but independent samples, *ε*_*Xj*_ and *ε*_*Yj*_ can be considered as mutually independent. Finally, although they must be estimated from the data, it is common practice to assume that the variances $\sigma_{Xj}^{2}$ and $\sigma_{Yj}^{2}$ are known. We will refer to the various assumptions and simplifications made in Section 2.1 as the ‘Two‐Sample Assumptions’ (TSA).

We now state some additional assumptions that are commonly utilised by the IVW or MR‐Egger approaches for causal inference (*vis* estimation of *β*), based on models (6) and (7) and the TSA. All of these assumptions are re‐stated for clarity in Table 1.

**Table 1:** Assumptions used in two‐sample MR analyses.

Assumption	Description
**IV assumptions**	
IV1	*γ*_*j*_ > 0: SNP predicts exposure (not through *U*).
IV2	*ψ*_*j*_ = 0: No SNP‐confounder association.
IV3	*α*_*j*_ = 0: No residual SNP‐outcome association
	after controlling for *X* and *U*.
**TSA**	
TSA1	Models (6) and (7) hold for population 1 and 2.
TSA2	*ε*_*Xj*_ in (6) and *ε*_*Yj*_ (7) independent
	of each other and any other terms.
TSA3	$\sigma_{Xj}^{2}$ and $\sigma_{Yj}^{2}$ in (6) and (7) known.
**NOME assumption**	$\sigma_{Xj}^{2}$ = 0: Negligible uncertainty in
	SNP‐exposure assxociation.
**InSIDE assumption (under IV2)**	
General InSIDE	Sample covariance cov $(\alpha_{j},\gamma_{j})\to$ 0 as L→∞.
Perfect InSIDE	Sample covariance cov(*α*_*j*_,*γ*_*j*_) = 0 for data at hand.
**VIS assumption (under IV2)**	*γ*_*j*_ ≠ *γ*_*i*_ for some *i* and *j* not equal to i
	Some variation in instrument strengths present.

### 2.2 Additional assumptions

#### 2.2.1 The NOME assumption

A pragmatic assumption from the MR literature is to assume $\sigma_{Xj}^{2}$ is small enough to be treated as zero. This would be strictly true if *N*_1_ were infinite, but is frequently reasonable because large study sizes (often over 100 000) are now common and rising year‐on‐year. This is referred to as the NO Measurement Error (NOME) assumption in Bowden et al 17.

#### 2.2.2 The InSIDE assumption

If assumptions IV2 and IV3 are satisfied for variant *j*(*α*_*j*_ = *ψ*_*j*_ = 0), then the total pleiotropic effect of variant *j* on the outcome is also zero. However, their violation does not necessarily preclude valid causal inference: consistent estimation of the causal effect is still possible if the magnitude of the pleiotropy in equation (7), *α*_*j*_ + *k*_*y*_ *ψ*_*j*_, is independent of the SNP‐exposure associations, *γ*_*j*_+ *k*_*x*_ *ψ*_*j*_. This was first identified as a crucial assumption for causal inference in the econometrics literature by Kolesar et al 18 and was independently derived for use in MR by Bowden et al 9, who termed it the InSIDE assumption (Instrument Strength Independent of Direct Effect). Because *ψ*_*j*_ is a common factor of both the instrument strength and pleiotropy terms, by far the most natural way to imagine that InSIDE could hold (even in principle) is if IV2 holds (*ψ*_*j*_ = 0) and the magnitude of the pleiotropy not via *U*(*α*_*j*_) is independent of *γ*_*j*_ across the instruments. We will therefore adopt this convention when referring to InSIDE from now on.

If the InSIDE assumption is satisfied then the sample covariance of the *α*_*j*_s and *γ*_*j*_s will tend to zero as the number of variants, *L*, increases. We will refer to this as ‘general ${}^{\prime }$ InSIDE. However, if the sample covariance of the *α*_*j*_s and *γ*_*j*_s is *exactly* zero for the data at hand, we will say that InSIDE is ‘perfectly ${}^{\prime }$ satisfied.

#### 2.2.3 The VIS assumption

If the SNP‐exposure association parameter estimates ${\hat{\gamma }}_{1},\ldots ,{\hat{\gamma }}_{L}$ were all identical, then their sample variance would be zero. If this were the case, it would rule out using the ${\hat{\gamma }}_{j}$s as an explanatory variable in any regression model (specifically MR‐Egger regression). We therefore define the VIS (Variation in Instrument Strength) assumption as being satisfied when the aforementioned sample variance of the true parameter values *γ*_1_,…,*γ*_*L*_ is non‐zero.

#### 2.2.4 NOME, InSIDE and VIS under a weighted analysis

In practice when implementing the IVW and MR‐Egger regression approaches, the analysis has traditionally been weighted by the standard error of the SNP‐outcome association estimates, *σ*_*Yj*_, to improve efficiency. For consistency, this is the approach we will subsequently follow. In order to define the NOME, InSIDE and VIS assumptions within the context of this weighted analysis (or any other weighted analysis), it is necessary to divide the relevant parameter by the chosen weight. For example, for VIS to be satisfied in a analysis weighted by *σ*_*Yj*_, we require some variation between the weighted instrument strength terms *γ*_*j*_/*σ*_*Yj*_. Similar transparent modifications would also be required for NOME and InSIDE.

## 3 Mendelian randomization under violations of IV3

In this section, we discuss the motivation for using the meta‐analytical methods of IVW and MR‐Egger regression, when all the assumptions outlined in Table 1 are satisfied except IV3. We will therefore use the following simplified versions of models (6) and (7) as our starting point:

(8) $$\begin{array}{ccc} \text{Data generating model under:}\;\; & \text{IV1‐2, TSA, NOME, InSIDE and VIS} & \\ \text{SNP‐exposure:}\;{\hat{\gamma }}_{j} & =\gamma_{j} \end{array}$$

(9) $$\text{SNP‐outcome:}\;\;\;\;\;\;{\hat{\Gamma }}_{j}=\alpha_{j}+\beta \gamma_{j}+\varepsilon_{Yj},\;\mathrm{var}(\varepsilon_{Yj})=\sigma_{Yj}^{2}$$

In order to easily discuss violations of IV3, we define the sample mean and variance of the *α* terms as *μ*_*α*_ and $\sigma_{\alpha }^{2}$, respectively. Specifically, we shall investigate four important special cases of model (9) that are defined and differentiated by the chosen values of *μ*_*α*_ and $\sigma_{\alpha }^{2}$. Under model (9), the *α*_*j*_ term is the sole source of additional variation in the SNP‐outcome association estimates (given the SNP‐exposure estimates), apart from the residual error term *ε*_*Yj*_. There may of course be factors inducing additional heterogeneity in the SNP‐outcome associations other than pleiotropy, so in practice we can think of *α*_*j*_ as representing the combination of all these sources. For simplicity, we will continue to refer to this as pleiotropy.

We start by assuming that IV3 actually holds for all variants in model (9), which is consistent with *α*_*j*_ = *μ*_*α*_ = $\sigma_{\alpha }^{2}$ = 0. We call this case (a) and it is the natural starting point for an MR analysis. In this case, model (9) can be simplified to the following linear model with no intercept:

(10) $$\frac{{\hat{\Gamma }}_{j}}{\sigma_{Yj}}=\frac{\beta \gamma_{j}}{\sigma_{Yj}}+\varepsilon_{j},\;\mathrm{var}(\varepsilon_{j})=1.$$

Both sides of the equation are divided by *σ*_*Yj*_ in order to make the variance homoscedastic and clarify the mechanics of model fitting under a weighted analysis. An overall estimate can be calculated from model (10) using standard regression theory, which yields the well known meta‐analytic formulae:

(11) $${\hat{\beta }}_{IVW}=\frac{\overset{L}{\underset{j=1}{\sum }}w_{j}{\hat{\beta }}_{j}}{\overset{L}{\underset{j=1}{\sum }}w_{j}},\;\;w_{j}=1/\mathrm{var}({\hat{\beta }}_{j})=\gamma_{j}^{2}/\sigma_{Yj}^{2}.$$

In the MR context, this is referred to as the IVW estimate 6, 12. Formula (11) can alternatively be derived by calculating a weighted average of the set of ratio estimates of causal effect, ${\hat{\beta }}_{j}={\hat{\Gamma }}_{j}/\gamma_{j}$, obtained using each SNP in turn, where the weight given to ${\hat{\beta }}_{j}$ is the inverse of its variance, $\sigma_{Yj}^{2}/\gamma_{j}^{2}$, as in a fixed effect meta‐analysis.

It is precisely assumptions IV2 and IV3 that dictate the intercept in (10) should be constrained to zero. The NOME assumption dictates the simple form for $\mathrm{var}({\hat{\beta }}_{j})$ because this means that the denominator of ${\hat{\beta }}_{j}$ can be treated as a constant (even though in truth it is the random variable ${\hat{\gamma }}_{j}$).

### 3.1 Testing and accounting for balanced pleiotropy

The variance of the IVW estimate under case (a) (the fixed effect model), is given by:

(12) $$Var({\hat{\beta }}_{IVW})=\frac{1}{\overset{L}{\underset{j=1}{\sum }}w_{j}}.$$

The value of 1 the numerator in (12) is a direct consequence of the fact that the variance of the residual error in model (10) is equal to 1. As suggested by Del Greco et al 16, the data can be scrutinised to assess this assumption by calculating Cochran ${}^{\prime }$s *Q* statistic with respect to the *L* IV estimates as follows:

(13) $$Q=\overset{L}{\underset{j=1}{\sum }}{\left(\frac{{\hat{\Gamma }}_{j}}{\sigma_{Yj}}-\frac{{\hat{\beta }}_{IVW}\gamma_{j}}{\sigma_{Yj}}\right)}^{2}=\overset{L}{\underset{j=1}{\sum }}w_{j}{\left({\hat{\beta }}_{j}-{\hat{\beta }}_{IVW}\right)}^{2}.$$

If true, *Q* follows a *χ* ^2^ distribution on *L*‐1 degrees of freedom. If the *L* estimates exhibit over‐dispersion or heterogeneity (as will often happen) then, under model (9), this must be due to pleiotropy and an extension to the basic model is required. The most natural first extension, we argue, would be to allow for ‘balanced ${}^{\prime }$ pleiotropy. That is, we relax IV3 to allow non‐zero *α*_*j*_s but assume they have zero mean (e.g. *μ*_*α*_= 0, $\sigma_{\alpha }^{2}>$ 0). We call this case (b) and re‐write model (10) as:

(14) $$\frac{{\hat{\Gamma }}_{j}}{\sqrt{\sigma_{Yj}^{2}+\sigma_{\alpha }^{2}}}=\frac{\beta \gamma_{j}}{\sqrt{\sigma_{Yj}^{2}+\sigma_{\alpha }^{2}}}+\varepsilon_{j},\;\mathrm{var}(\varepsilon_{j})=1.$$

Model (14) is justified for any *L* under Perfect InSIDE, because this allows pleiotropy to be treated as independent residual error with respect to the explanatory variable *γ*_*j*_. It is only strictly valid under General InSIDE as L→∞. Fitting model (14) is equivalent to performing a standard additive random effects meta‐analysis 19, 20. That is, we can obtain a new IVW point estimate and variance for the causal effect under model (14) by applying formulae (11) and (12) and replacing *w*_*j*_ = $\gamma_{j}^{2}$/ $\sigma_{Yj}^{2}$ with $w_{j}^{*}$ = $\gamma_{j}^{2}$/( $\sigma_{Yj}^{2}+\sigma_{\alpha }^{2}$) using a plug‐in estimate for $\sigma_{\alpha }^{2}$(for example the DerSimonian and Laird estimate 19. We will refer to the IVW estimate obtained by fitting model (14) as ${\hat{\beta }}_{IVW}^{ARE}$. The two IVW estimates ${\hat{\beta }}_{IVW}$ and ${\hat{\beta }}_{IVW}^{ARE}$ do differ, because under the latter the variance component $\sigma_{\alpha }^{2}$ is used to re‐weight the contribution of the *j*th IV estimate. The variance of ${\hat{\beta }}_{IVW}^{ARE}$ is additionally inflated via $w_{j}^{*}$ to account for heterogeneity due to pleiotropy. When a large value of $\sigma_{\alpha }^{2}$ is estimated from the data, this will have the effect of evening out the weight given to each ratio estimate in the analysis by making them a less direct function of each estimate ${}^{\prime }$s precision.

#### 3.1.1 Multiplicative random effects models

The following multiplicative random effects model 21, 22 could instead be applied to the summary data estimates in the presence of observed heterogeneity:

(15) $$\frac{{\hat{\Gamma }}_{j}}{\sigma_{Yj}}=\frac{\beta \gamma_{j}}{\sigma_{Yj}}+\frac{\varphi^{1/2}}{\sigma_{Yj}}\varepsilon_{j},\;\mathrm{var}(\varepsilon_{j})=1.$$

If adopted, model (15) returns the same point estimate for *β* as under the fixed effect IVW model, ${\hat{\beta }}_{IVW}$. That is, the variance component $\sigma_{\alpha }^{2}$ is not allowed to influence its point estimate. However, the addition of the scale parameter *φ* ^1/2^ allows the variance of ${\hat{\beta }}_{IVW}$ to increase when heterogeneity is detected. Specifically, *φ* is estimated from the variance of the residual error using ${\hat{\varphi }}_{IVW}=\frac{Q}{L-1}$ where *Q* is defined in (13). The variance of the IVW estimate under model (15) is then set to

$$\mathrm{var}({\hat{\beta }}_{IVW})=\frac{{\hat{\varphi }}_{IVW}}{\overset{L}{\underset{j=1}{\sum }}w_{j}},$$

so that it is ${\hat{\varphi }}_{IVW}$ times the variance under the fixed effect model ‐ case (a).

If the additive pleiotropy model (14) is actually correct then the residual error of the multiplicative pleiotropy model (15) is miss‐specified, because the constant term *φ* ^1/2^ should be replaced with $\sqrt{1+\frac{\sigma_{\alpha }^{2}}{\sigma_{Yj}^{2}}}$, which will usually result in a small loss of efficiency. One reason to prefer the multiplicative model in the general meta‐analysis context, is that ${\hat{\beta }}_{IVW}$ is known to be more robust to small study bias than ${\hat{\beta }}_{IVW}^{ARE}$ 15. We will subsequently show that the same principle holds true in the MR context, when ‘directional ${}^{\prime }$ pleiotropy is present in the data, a notion to which we now turn our attention.

### 3.2 Testing and accounting for directional pleiotropy

‘Directional’ pleiotropy occurs when the mean value of the pleiotropy distribution, *μ*_*α*_, is non‐zero 9. We will show how this can be addressed by performing MR‐Egger regression. We first consider the special case where *μ*_*α*_ is non‐zero but $\sigma_{\alpha }^{2}$ = 0. This means that *α*_*j*_= *μ*_*α*_ for all *j* in model (7). Although this appears highly unlikely, it is actually what MR‐Egger regression (as proposed in 9) assumes in its simplest form. We call this case (c), which can be written as

(16) $$\frac{{\hat{\Gamma }}_{j}}{\sigma_{Yj}}=\frac{\mu_{\alpha }}{\sigma_{Yj}}+\beta \frac{\gamma_{j}}{\sigma_{Yj}}+\varepsilon_{j},\;\mathrm{var}(\varepsilon_{j})=1$$

MR‐Egger regression fits model (16) under case (c) to give an estimate for the intercept *μ*_*α*_ and causal effect *β*. In order to distinguish them from the IVW estimate, we will refer to the MR‐Egger estimate for the causal effect *β* as ${\hat{\beta }}_{1E}$ and the mean pleiotropic effect *μ*_*α*_ as ${\hat{\beta }}_{0E}$, where

$$\left(\begin{array}{c} {\hat{\beta }}_{0E}\\ {\hat{\beta }}_{1E} \end{array}\right)={\left(Z^{T}Z\right)}^{-1}Z^{T}\hat{\Gamma },\;\text{for}\;Z=\left(\begin{array}{cc} \sigma_{Y1}^{-1} & \gamma_{1}\sigma_{Y1}^{-1}\\ \sigma_{Y2}^{-1} & \gamma_{2}\sigma_{Y2}^{-1}\\ . & .\\ . & .\\ . & .\\ \sigma_{YL}^{-1} & \gamma_{L}\sigma_{YL}^{-1} \end{array}\right),\;\hat{\Gamma }=\left(\begin{array}{c} {\hat{\Gamma }}_{1}\sigma_{Y1}^{-1}\\ {\hat{\Gamma }}_{2}\sigma_{Y2}^{-1}\\ .\\ .\\ .\\ {\hat{\Gamma }}_{L}\sigma_{YL}^{-1} \end{array}\right).$$

The variance of the MR‐Egger estimate is equal to the lower diagonal element of **(*Z*^*T*^*Z*)^ − 1^** but when *σ*_*Y*1_,…,*σ*_*YL*_ are all exactly equal, this reduces to

(17) $$\mathrm{var}({\hat{\beta }}_{1E})=\frac{1}{\overset{L}{\underset{j=1}{\sum }}{\left(\frac{{\hat{\gamma }}_{j}-\bar{\hat{\gamma }}}{\sigma_{Yj}}\right)}^{2}}.$$

This simple formula indicates that the variance of ${\hat{\beta }}_{1E}$ is essentially dictated by the variance of the SNP‐exposure associations. It also reinforces the importance of the VIS assumption for MR‐Egger: its causal estimate is simply undefined when no such variation exists.

Case (c) allows for directional pleiotropy but assumes that the pleiotropic effect is the same across all variants. This means that pleiotropy induces bias but no additional heterogeneity, which implies that the residual error about model (16) equals 1. We can test the plausibility of this assumption via a simple adaptation of the $Q^{\prime }$ statistic due to Rücker et al 14 to our context:

(18) $$Q^{\prime }=\overset{L}{\underset{j=1}{\sum }}\frac{1}{\sigma_{Yj}^{2}}{\left\{{\hat{\Gamma }}_{j}-\left({\hat{\beta }}_{0E}+{\hat{\beta }}_{1E}\gamma_{j}\right)\right\}}^{2}=\overset{L}{\underset{j=1}{\sum }}w_{j}{\left({\hat{\beta }}_{j}-\frac{{\hat{\beta }}_{0E}}{\gamma_{j}}-{\hat{\beta }}_{1E}\right)}^{2},$$

where $w_{j}=\frac{\gamma_{j}^{2}}{\sigma_{Yj}^{2}}$ as before. If case (c) is correct then $Q^{\prime }$ should follow a *χ* ^2^ distribution with *L*‐2 degrees of freedom. If a non‐zero intercept ${\hat{\beta }}_{0E}$ is estimated and the $Q^{\prime }$ statistic is significantly larger than *L* − 2, a more reasonable model might be that directional pleiotropy is inducing bias **and** additional heterogeneity into the MR summary data estimates ( $\mu_{\alpha }\neq 0,\sigma_{\alpha }^{2}>$0). We call this case (d) and re‐write model (16) accordingly as an additive random effect model 14:

(19) $$\frac{{\hat{\Gamma }}_{j}}{\sqrt{\sigma_{Yj}^{2}+\sigma_{\alpha }^{2}}}=\frac{\mu_{\alpha }}{\sqrt{\sigma_{Yj}^{2}+\sigma_{\alpha }^{2}}}+\frac{\beta \gamma_{j}}{\sqrt{\sigma_{Yj}^{2}+\sigma_{\alpha }^{2}}}+\varepsilon_{j},\;\mathrm{var}(\varepsilon_{j})=1.$$

As for case (b), model (19) is valid for any *L* under perfect InSIDE and as L→∞ under General InSIDE. Updated estimates ${\hat{\beta }}_{0E}^{ARE}$ and ${\hat{\beta }}_{1E}^{ARE}$ could be obtained by changing the definition of the design matrix ***Z*** so that the *j*th row is equal to

$$\left(\frac{1}{\sqrt{\sigma_{Yj}^{2}+\sigma_{\alpha }^{2}}},\frac{\gamma_{j}}{\sqrt{\sigma_{Yj}^{2}+\sigma_{\alpha }^{2}}}\right)$$

and where $\sigma_{\alpha }^{2}$ is substituted with a suitable estimate. For the reasons already outlined in the case of the IVW model, we have so far preferred not to use the additive pleiotropy model (19) in practice to fit MR‐Egger regression. We have instead opted to preserve the original fixed effect MR‐Egger point estimates, and to scale up their variance if heterogeneity is detected, by fitting the multiplicative model:

(20) $$\frac{{\hat{\Gamma }}_{j}}{\sigma_{Yj}}=\frac{\mu_{\alpha }}{\sigma_{Yj}}+\beta \frac{\gamma_{j}}{\sigma_{Yj}}+\frac{\varphi^{1/2}}{\sigma_{Yj}}\varepsilon_{j},\;\mathrm{var}(\varepsilon_{j})=1.$$

Under model (20), additional heterogeneity can be taken into account by estimating *φ* via ${\hat{\varphi }}_{E}=Q^{\prime }/(L-2)$ and scaling up the variance of the MR‐Egger estimates to be ${\hat{\varphi }}_{E}$ times their value under case (c).

### 3.3 When will the IVW and MR‐Egger methods be unbiased?

Table 2 attempts to clarify the additional assumptions that are required for unbiased estimation of *β* by the IVW and MR‐Egger methods under cases (a) to (d). We use the word ‘additional’ to distinguish them from baseline assumptions (which we define as TSA, NOME and IV2) as well as those that are implied by each case (such as IV3 in case (a)). If a specific case automatically invalidates a method, so that unbiased estimation is not possible in general, we mark it with an ‘ × ’.

**Table 2:** Requirements for unbiased estimation of *β* by the IVW and MR‐Egger regression approaches for special cases (a) to (d).

Case (model) baseline & implied	Pleiotropy distribution		Unbiased estimation of *β* possible under additional assumptions?	
assumptions	*μ*_*α*_	$\sigma_{\alpha }^{2}$	IVW	MR‐Egger
(a): No pleiotropy	0	0	IV1	VIS
(Fixed effect IVW)				
TSA,NOME,IV2				
IV3, Perfect InSIDE				
(b): Balanced pleiotropy	0	> 0	IV1, General InSIDE	VIS, General InSIDE
(Random effect IVW)				
TSA,NOME,IV2				
(c): Directional pleiotropy	≠ 0	0	×	VIS
(Fixed effect MR‐Egger)				
TSA,NOME,IV2				
Perfect InSIDE				
(d): Directional pleiotropy	≠ 0	> 0	×	VIS, General InSIDE
(Random effect MR‐Egger)				
TSA,NOME, IV2				

Under case (a) (no pleiotropy), assumptions IV2 and IV3 hold which means that Perfect InSIDE is satisfied because cov(0,*γ*_*j*_) ≡  0. The IVW method therefore only requires IV1 to hold and MR‐Egger only requires VIS to hold in order to be unbiased.

Under case (b) (balanced pleiotropy), both methods are consistent (asymptotically unbiased as L→∞) under the General InSIDE assumption because heterogeneous pleiotropy contributes to the residual error. Under cases (c) and (d) (directional pleiotropy), the IVW estimate cannot consistently estimate *β* in general because it does not adjust for a non‐zero mean pleiotropic effect. Under case (c), Perfect InSIDE is satisfied because cov(*μ*_*α*_,*γ*_*j*_)= 0. The only additional assumption required by MR‐Egger for unbiased estimation is VIS. Under case (d), MR‐Egger requires both VIS and General InSIDE to be consistent.

## 4 Can we navigate between models using Rücker's framework?

Moving from the underlying model in case (a) to those in (b), (c) and finally (d) represents a natural way to progressively relax assumption IV3 using heterogeneity statistics when performing MR. Following the approach of Rücker et al in the general meta‐analysis context, we would start by assuming all genetic variants are valid ‐ case (a) ‐ but move to case (b) if Cochran's *Q* is sufficiently extreme with respect to a $\chi_{L-1}^{2}$ distribution, in order to allow for balanced pleiotropy. We would then assess whether directional pleiotropy is present by fitting model (c) and calculate the heterogeneity about this model via Rücker's $Q^{\prime }$. If the difference $Q-Q^{\prime }$ is sufficiently extreme with respect to a $\chi_{1}^{2}$ distribution, we would infer that directional pleiotropy is an important factor, and adopt model (c). Finally, we would assess whether $Q^{\prime }$ is sufficiently extreme with respect to a $\chi_{L-2}^{2}$ distribution. If so, we would opt for model (d) to account for the fact that pleiotropy is inducing heterogeneity and bias.

Unfortunately, this framework does not seamlessly translate to the MR setting, because IVW model (10) is not strictly nested within MR‐Egger model (16). This means that *Q* ‐ *Q′* is only approximately $\chi_{1}^{2}$ distributed. *Q′* may in fact be larger than *Q*, making *Q‐Q′* negative. The equivalent IVW and Egger regression models assumed by Rücker et al, are nested so that the equivalent *Q‐Q′* difference has the required distribution.

Therefore, we can only informally use a *Q‐Q′* to decide whether MR‐Egger regression represents a better fit than IVW. We therefore suggest the user considers moving from IVW model's (a) and (b) to MR‐Egger model's (c) and (d) when *Q‐Q′* is large and positive, and the MR‐Egger intercept parameter estimate is sufficiently far from zero and precise.

### 4.1 An illustrative example

**Figure 3:**
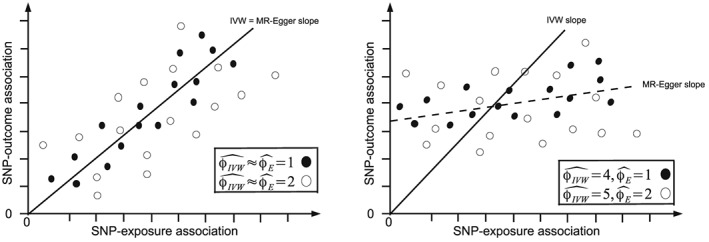
Scatter plot of SNP‐outcome versus SNP‐exposure estimates for four fictional MR analyses. Left: under cases (a) (solid black dots) and (b) (hollow black dots). Right: under cases (c) (solid black dots) and (d) (hollow black dots).

Figure 3 (left) shows an illustrative scatter plot of SNP‐outcome and SNP‐exposure associations consistent with case (a) (solid black dots, no pleiotropy) and case (b) (hollow black dots, balanced pleiotropy). Under both cases (a) and (b) the points yield a regression line perfectly through the origin. This means that the IVW and MR‐Egger causal estimates would be identical ( ${\hat{\beta }}_{IVW}$ = ${\hat{\beta }}_{1E}$). Under case (a), ${\hat{\varphi }}_{IVW}$ and ${\hat{\varphi }}_{E}$ would be approximately equal to each other and to 1, and under case (b) ${\hat{\varphi }}_{IVW}$ and ${\hat{\varphi }}_{E}$ would be approximately equal to each other with a value greater than 1 (2 in this instance). For the solid black data, Cochran's *Q* would provide no evidence to support a move from the basic model (a) and the IVW estimate. For the hollow black data, Cochran's *Q* would suggest sticking with the IVW estimate, but to allow for over‐dispersion due to balanced pleiotropy by moving to model (b). Because the MR‐Egger estimate is identical to the IVW estimate, this means that ${\hat{\beta }}_{0E}$ = 0 and $Q-Q^{\prime }$ = 0. Therefore, no evidence exists to support moving to model (c) and the MR‐Egger estimate.

Figure 3 (right) shows an illustrative scatter‐plot of SNP‐outcome and SNP‐exposure associations consistent with case (c) (solid black dots, homogeneous directional pleiotropy) and case (d) (hollow black dots, heterogeneous directional pleiotropy). Under both cases (c) and (d) the points are consistent with a regression line with the same non‐zero intercept. Under case (c), ${\hat{\varphi }}_{E}$ is equal to 1 whereas ${\hat{\varphi }}_{IVW}$ is much larger (4 in this instance). Under case (d) ${\hat{\varphi }}_{E}$ is equal to 2 whereas ${\hat{\varphi }}_{IVW}$ is again larger (5 in this instance). Now, the solid black data would support a move from model (a) (and the IVW estimate) to model (c) (and the MR‐Egger estimate) because $Q,{\hat{\beta }}_{0E}$ and $Q-Q^{\prime }$ are ‘large’, but $Q^{\prime }$ on its own is not. The hollow black dots would additionally yield a large enough $Q^{\prime }$ in order to opt for model (d), thereby suggesting inference should be based on the MR‐Egger estimate accounting for heterogeneity and bias due to pleiotropy.

As in the general meta‐analysis context, heterogeneity statistics such as *Q* and *Q′* are only strictly valid under the assumption that the precision of SNP‐exposure and SNP‐outcome estimates are known, despite being estimated from the data. Likewise, their power to ‘detect’ heterogeneity will be small when the number of genetic variants, *L*, is small. This could mean that, even if model (d) were true, it may often not be possible to infer this by rejecting models (a), (b) and (c) at the historically popular 5% significance level. Furthermore, choosing a model (and by extension a causal estimate) that depends on the value of a statistical test (i.e ‘testimation’ 23,23) could also induce unwanted bias. Additional research is required to fully understand the operating characteristics of the schema suggested here in realistic data settings, especially the use of heterogeneity statistic estimators, before they can be confidently used in practice.

#### 4.1.1 A measure of relative fit

In Figure 3 (left), we see strong evidence that the IVW estimate is a reliable measure of causal effect. Because it is also guaranteed to be more precise, there is no reason whatsoever to prefer the MR‐Egger estimate. Conversely, in Figure 3 (right), we see strong evidence that the IV assumptions have been violated in a meaningful way and the MR‐Egger approach has more appeal. We therefore seek a statistic that will favour the IVW approach *a priori* unless MR‐Egger regression is a demonstratively better fit to the data. A simple statistic, *Q*_*R*_ encapsulates this reasoning, and is again closely connected to the work of Rücker et al 14. It simply measures the relative value of the residual heterogeneity under both the IVW and MR‐Egger methods:

(21) $$Q_{R}=\left(\frac{Q^{\prime }}{Q}\right)$$

*Q*_*R*_ will generally lie in the unit interval, but because the MR‐Egger and IVW models are non‐nested, in rare cases the inequality $0⩽Q^{\prime }⩽Q$ will not hold. In Figure 3 (left), we see that (under case (a) or (b)) *Q*_*R*_ is approximately equal to 1, which tells us that the IVW method is to be preferred over MR‐Egger. In Figure 3 (right), we see that *Q*_*R*_ is equal to 1/4 in case (c) and 2/5 in case (d), indicating that inferences from the MR‐Egger approach should be given due consideration. In the limit, if the IVW approach was an increasingly bad fit to the data relative to MR‐Egger, then *Q*_*R*_ would tend to zero and MR‐Egger should be the preferred choice.

## 5 Sensitivity analysis

### 5.1 Estimation under violations of IV2 and IV3

In Section 3, we assumed that IV2 held to demonstrate how established meta‐analytic methods can, in theory, be transparently adapted to the MR setting. We now explore, from a theoretical standpoint, how IV2 and IV3 violation distort the causal estimand identified by the IVW and MR‐Egger approaches. To do this, we return to and assume models (8) and (9) hold, set *κ*_*x*_ = *κ*_*y*_ = 1, but allow the *ψ*_*j*_ terms to be non‐zero. When this is the case, the true estimand for the causal effect identified by variant *j* using the ratio method is:

(22) $$\beta_{j}=\beta +\frac{\alpha_{j}+\psi_{j}}{\gamma_{j}+\psi_{j}}$$

We now make an additional simplifying assumption that the SNP‐outcome standard error is constant across variants (*σ*_*Yj*_ = *σ*_*Y*_). When this is true, the fixed effect IVW estimand is equal to

(23) $$\beta_{IVW}=\beta +\frac{\overset{L}{\underset{j=1}{\sum }}(\gamma_{j}+\psi_{j})(\alpha_{j}+\psi_{j})}{\overset{L}{\underset{j=1}{\sum }}{\left(\gamma_{j}+\psi_{j}\right)}^{2}}=\beta +\frac{\text{cov}(\gamma_{j}+\psi_{j},\alpha_{j}+\psi_{j})+E[\gamma_{j}+\psi_{j}]E[\alpha_{j}+\psi_{j}]}{\mathrm{var}(\gamma_{j}+\psi_{j})+E{\left[\gamma_{j}+\psi_{j}\right]}^{2}},$$

and the fixed effect MR‐Egger estimand is equal to

(24) $$\beta_{1E}=\beta +\frac{\text{cov}\left(\gamma_{j}+\psi_{j},\alpha_{j}+\psi_{j}\right)}{\mathrm{var}(\gamma_{j}+\psi_{j})},$$

where E[.], cov(.) and var(.) refer to sample expectations, covariances and variances, respectively. If IV2 were satisfied (*ψ*_*j*_ = 0 for all *j*,) then the MR‐Egger estimand tends to *β* as *L* grows large under General InSIDE because the numerator of the bias term in (24) will tend to zero. If, in addition, the sample mean of the *α*_*j*_s were zero (General InSIDE plus balanced pleiotropy) then the IVW estimand would also tend to *β* as *L* grew large because the numerator of the bias term in (23) would tend to zero.

**Figure 4:**
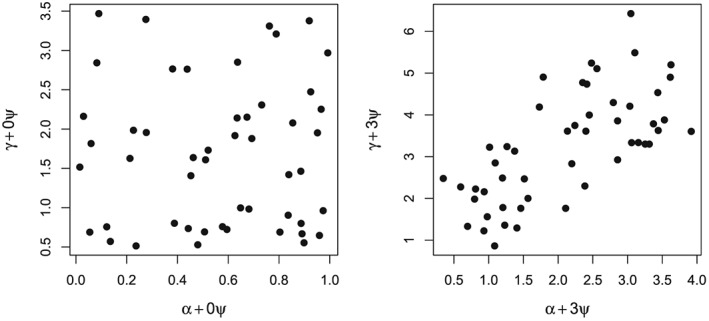
Left: Scatter plot of *γ* + 0 × *ψ* versus *α* + 0 × *ψ* parameters, where *α* and *γ* are generated to satisfy Perfect InSIDE. Right: Scatter plot of *γ* + 3 × *ψ* versus *α* + 3 × *ψ*.

**Figure A.1:**
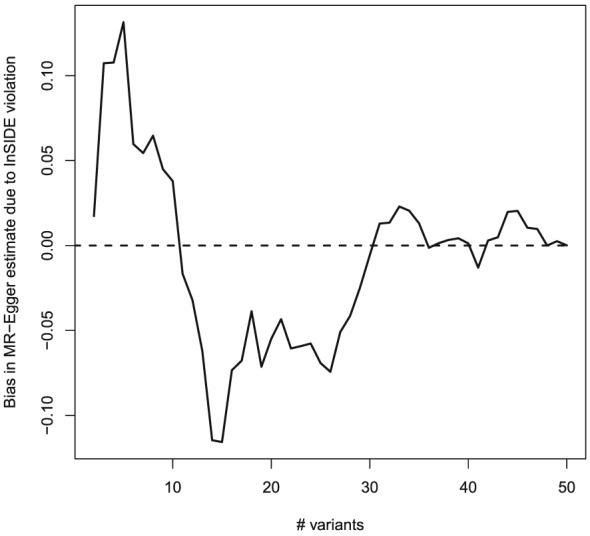
Theoretical bias of the MR‐Egger estimand using the parameter values of Table A1, as the number of included SNPs is increased sequentially from 1:2 to 1:50. Note: the parameters of Table A1 are generated independently so that General InSIDE is satisfied under IV2 for any subset of the 50 SNPs, but that Perfect InSIDE is satisfied when all 50 are considered.

In order to investigate the bias of the IVW and MR‐Egger estimators under increasing IV2 violation, we explore the value of their underlying estimands, by firstly generating three independent fixed vectors of *L* = 50 *α*,*γ* and *ψ* parameters with means 1/2, 2 and 1/2, respectively. For clarity, care was taken to ensure that the sample covariance of all 50 *α* and *γ* parameters was very close to zero, so that Perfect InSIDE is satisfied across all variants when the *ψ*_*j*_ terms are 0 (IV2 satisfied). The full parameter vectors are given in Table A1 in the Appendix. In addition, Figure A.1 in the Appendix plots the bias term of the MR‐Egger causal estimand in equation  (24) under IV2 when the included set of instruments is increased sequentially from 2 to 50. This is shown to illustrate the point that, even when *α*_*j*_ and *γ*_*j*_ are generated by independent processes (i.e. under General InSIDE), a sufficiently large number of variants is needed to ensure that their scaled sample covariance (i.e. the MR‐Egger bias term) settles at 0. In Figure 4 (left), we plot the vector *γ* versus the vector *α* to show the instrument strength and pleiotropic effect parameters are uncorrelated under IV2 and Perfect InSIDE. We label the axes with the addition of ‘0 × *ψ*’ to stress why this is the case.

In Figure 4 (right), we plot *γ* + 3*ψ* versus *α* + 3*ψ* to show the instrument strength and direct pleiotropic effect parameters when IV2 and InSIDE are violated. In this case, non‐zero *ψ* parameters induce a strong positive correlation between the combined instrument strength and pleiotropy terms. We now define the sensitivity parameter

$$\rho_{A}=\text{cor}(\gamma +A\psi ,\alpha +A\psi ).$$

That is, *ρ*_*A*_ equals the correlation between the instrument strength and direct effect due to pleiotropy when *ψ*_*j*_ is set to *A* × *ψ*_*j*_. In Figure 4 (left), *ρ*_0_ = 0 and in Figure 4 (right), *ρ*_3_= 0.62.

**Figure 5:**
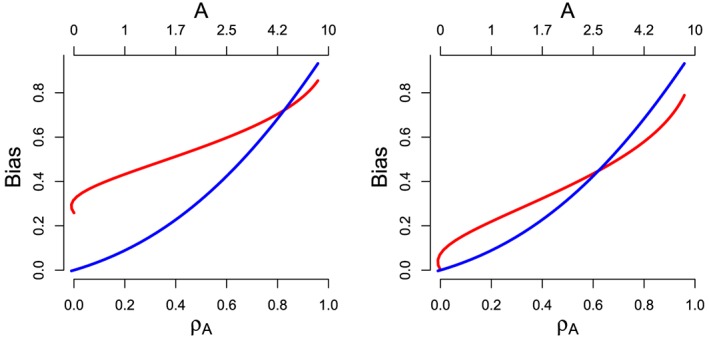
Theoretical bias in the MR‐Egger estimand *β*_1*E*_ (blue) and IVW estimand *β*_*IVW*_ (red) as a function of InSIDE violation via *ρ*_*A*_ for directional pleiotropy (left) and balanced pleiotropy (right). [Colour figure can be viewed at wileyonlinelibrary.com]

In Figure 5 (left), we plot the value of *β*_*IVW*_ and *β*_1*E*_ in equations (23) and (24) as a function of *A* and *ρ*_*A*_ for *β* = 0. Their values can therefore be interpreted as a bias. Because the mean pleiotropic effect is non‐zero, *β*_*IVW*_ is biased even under InSIDE, whereas *β*_1*E*_ is not. However, we see that *β*_1*E*_ is more strongly affected by violations of InSIDE than *β*_*IVW*_. When A⩾ 4.2 and $\rho_{A}⩾$ 0.8, *β*_1*E*_ is actually further away from the truth than *β*_*IVW*_. In Figure 5 (right), we plot the value of the IVW and MR‐Egger estimands as in Figure 5 (left) when *α*_*j*_ is replaced with $\alpha_{j}-\bar{\alpha }$, in order to make the pleiotropy balanced at *A*=0. Consequently, when *ρ*_0_= 0, *β*_*IVW*_ is unbiased. Furthermore, the IVW estimand is less biased than the *β*_1*E*_ for A⩾ 2.5 and $\rho_{A}⩾$ 0.65.

### 5.2 Fixed versus additive random effects for IVW

In Section 3, we cautioned against using the additive random effects IVW estimate ${\hat{\beta }}_{IVW}^{ARE}$ as opposed to the fixed effect estimate ${\hat{\beta }}_{IVW}$ when directional pleiotropy is present. In order to explain the reason for our caution, we plot the value of the additive random effects IVW estimand, $\beta_{IVW}^{ARE}$ versus its fixed effect counterpart (at *β*=0) using our fixed parameter constellation under increasing violations of the InSIDE assumption as before.

**Figure 6:**
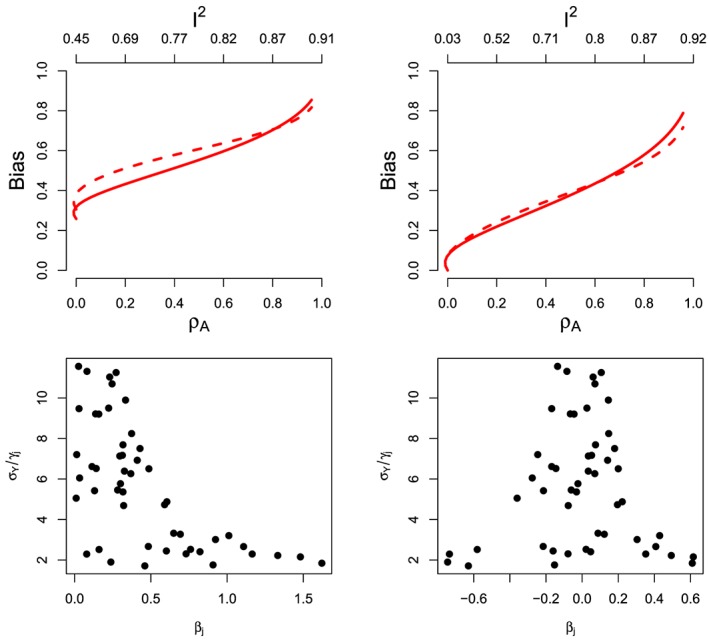
Top: Theoretical bias of the fixed effect estimand *β*_*IVW*_ (red solid line) and additive random effect estimand $\beta_{IVW}^{ARE}$ (red dashed line) as a function of InSIDE violation in the case of directional pleiotropy (left) and balanced pleiotropy (right). Bottom: Funnel plots of the inverse standard errors versus their causal estimands *β*_*j*_ at *ρ*=0 and *σ*_*Y*_ = 0.3 for directional pleiotropy (left) and balanced pleiotropy (right). [Colour figure can be viewed at wileyonlinelibrary.com]

We calculated $\beta_{IVW}^{ARE}$ using equation (11) by plugging in the estimand for *β*_*j*_ from equation (22) and using random effects weights $w_{j}^{*}$= ${\left(\gamma_{j}+A\psi_{j}\right)}^{2}/(\sigma_{Yj}^{2}+\sigma_{\alpha }^{2})$. In order to specify these weights, we set *σ*_*Yj*_ equal to a constant value of 0.3, and then used the DerSimonian and Laird method to estimate $\sigma_{\alpha }^{2}$ for each given value of A. Figure 6 shows the results for the two previously discussed scenarios of directional pleiotropy using (*α*,*γ*,*ψ*) and balanced *α* pleiotropy using ( $\alpha -\bar{\alpha },\gamma ,\psi$). In order to aid interpretation, the *x*‐axis includes scales for *ρ*_*A*_ and, additionally, the amount of heterogeneity as measured by Higgins' *I* ^2^ statistic 25: (*Q* − (*L* − 1))/*Q*.

Under the directional pleiotropy scenario (Figure 6, top‐left), both $\beta_{IVW}^{ARE}$ and *β*_*IVW*_ are biased even when the InSIDE assumption is satisfied (*ρ*_*A*_= 0). The directional pleiotropy manifests itself as heterogeneity about the fixed effect IVW estimate (as measured by Cochran's *Q* statistic), and at *ρ*_*A*_=0, *I* ^2^=0.45. As *ρ* increases *β*_*IVW*_ is more robust than $\beta_{IVW}^{ARE}$, staying closer to the true value of zero for $\rho_{A}⩽$ 0.8. This occurs because the observed heterogeneity is caused, disproportionately, by *β*_*j*_ terms emanating from variants with a weaker SNP‐exposure association, because their bias terms in equation (22) will (in general) be the largest.

When it detects heterogeneity, the additive random effects estimate compounds the problem by re‐weighting the ratio estimates more evenly across the board, thereby giving more weight to the weaker (and more biased) ratio estimates. The fixed effect estimate, and by extension the multiplicative random effects estimate, naturally gives ratio estimates from variants with weaker SNP‐exposure associations little weight in the analysis, regardless of whether heterogeneity is detected or not.

Under the balanced pleiotropy example (Figure 6, top‐right), both *β*_*IVW*_ and $\beta_{IVW}^{ARE}$ are unbiased at *ρ*_*A*_=0. At *ρ*_*A*_=0, *I* ^2^ is very close to zero also, indicating that almost all of the heterogeneity observed in the directional pleiotropy case discussed earlier at *ρ*_*A*_=0 was due to bias (i.e. non‐zero *μ*_*α*_). As *ρ*_*A*_ increases, *β*_*IVW*_ and $\beta_{IVW}^{ARE}$ remain close together. This is because there is no longer a directional trend in the *β*_*j*_s as a function of the *j*th SNP‐exposure association. In order to stress this point, Figure 6 (bottom) shows funnel plots of the inverse standard errors versus their causal estimands *β*_*j*_ at *ρ*_*A*_ = 0 under directional (bottom‐left) and balanced (bottom‐right) pleiotropy. Inverse standard errors used in the *y*‐axis are calculated under NOME. The funnel plot is used as a graphical tool to aid detection of small study bias in meta‐analysis 26 and has been transferred to the MR setting by Bowden et al 9. Only in the directional pleiotropy case will asymmetry be observed in the funnel plot. That is, imprecise causal effect estimates are not seen to ‘funnel’ in towards the more precise causal estimates equally from either side, but appear to be skewed in one particular direction. Therefore, funnel plot asymmetry is a sure sign that a large difference will exist between the fixed and additive random effects IVW estimates, as well as between the IVW and MR‐Egger estimates.

### 5.3 Estimation under violations of NOME

So far, in this paper, we have assumed that the NOME assumption is satisfied. In truth, however, NOME will never hold exactly because the variance of the SNP‐exposure association, $\sigma_{Xj}^{2}$ will always be positive, so that ${\hat{\gamma }}_{j}\neq \gamma_{j}$. In this context, we can think of IVW and MR‐Egger approaches as fitting regression models using an explanatory variable that is affected by measurement error. This is known to induce an attenuation in the subsequent effect estimates towards zero due to ‘regression dilution’ 27, 28. The implications of NOME violation on the two approaches is discussed at length in separate work by Bowden et al 17, but are summarised below.

For clarity, suppose that the pleiotropy distribution and additional assumptions defining either case (a) or (b) holds (Table 2 so that both the IVW and MR‐Egger methods yield unbiased estimates for *β* under NOME (i.e. when $\sigma_{Xj}^{2}$ = 0 for all *j*). When NOME is in fact violated for variant *j*( $\sigma_{Xj}^{2}>$ 0), the expected attenuation in its individual ratio estimate, ${\hat{\beta }}_{j}$, can be approximated by:

$$E[{\hat{\beta }}_{j}]\approx \beta \left(\frac{F_{j}-1}{F_{j}}\right),$$

where *F*_*j*_ = ${\hat{\gamma }}_{j}^{2}/\sigma_{Xj}^{2}$. Because it is a weighted average of ratio estimates, the expected attenuation in the IVW estimate, ${\hat{\beta }}_{IVW}$, can therefore be approximated by substituting *F*_*j*_ in the above formula with $\bar{F}=\sum_{j=1}^{L}w_{j}F_{j}/\sum_{j=1}^{L}w_{j}$, where *w*_*j*_ are as defined in (11). The attenuation in the IVW estimate can be alleviated by increasing the strength of each instrument through *F*_*j*_ so that IV1 is more strongly satisfied.

In contrast, the extent of attenuation in the MR‐Egger estimate, ${\hat{\beta }}_{1E}$, due to NOME violation is not governed by the same *F* statistic. It can instead be accurately gauged using a modification of the *I* ^2^ statistic applied directly to the entire set of SNP‐exposure associations (and referred to as $I_{GX}^{2}$ in 17 as below:

$$E[{\hat{\beta }}_{1E}]\approx \beta I_{GX}^{2},\;\text{where}\;I_{GX}^{2}=(Q_{GX}-(L-1))/Q_{GX}\;\text{for}\;Q_{GX}=\overset{L}{\underset{j=1}{\sum }}\frac{{\left({\hat{\gamma }}_{j}/\sigma_{Yj}-\overset{j_{¯}}{\hat{\gamma }}\right)}^{2}}{\sigma_{Xj}^{2}/\sigma_{Yj}^{2}},$$

where γ^¯ is the mean of the weighted ${\hat{\gamma }}_{j}$s. The $I_{GX}^{2}$ statistic measures the spread of variation in the estimated instrument strengths (the ${\hat{\gamma }}_{j}$s) relative to their average uncertainty. It lies between 0 and 1 but is generally reported as a percentage. An $I_{GX}^{2}$ close to 100% ensures that the attenuation will be minimal, and means that the VIS assumption is strongly satisfied.

In most circumstances, the extent of attenuation is likely to be far worse for MR‐Egger than for the IVW estimate. Bowden et al 17 employ the method of Simulation Extrapolation (SIMEX) 29, 30 to adjust the MR‐Egger estimate for this dilution. Briefly, this involves generating pseudo‐data sets based on the original summary data estimates, but under increasingly strong violations of the NOME assumption, to obtain a series of increasingly diluted parameter estimates. A statistical model is then fitted to this series in order to extrapolate back to the estimate that would have been obtained if NOME **had** been satisfied. In the next section, we show the SIMEX approach applied in practice to both the IVW and MR‐Egger estimates.

## 6 Assessing the causal role of plasma urate on CHD risk

Although traditional epidemiological studies have suggested that urate levels are associated with a range of cardiometabolic diseases, it is not certain whether the relationship is causal. Recently, Mendelian randomization has been applied to assess the possibility of a causal link 31, 32. Specifically, White and colleagues 32 conducted a two‐sample summary data MR analysis to assess the causal role of plasma urate levels in influencing cardiovascular disease risk. Summary data estimates of the association of 31 uncorrelated genetic variants with plasma urate concentration were obtained from the genome wide association studies catalogue http://www.genome.gov/gwastudies/ with *p*‐values less than 5 × 10^ − 7^. Estimates for the association of the 31 variants with cardiovascular disease risk were obtained from a seperate study population using CARDIoGRAM 3, currently the largest genetic association study measuring cardiovascular outcomes. Here, we repeat and extend the analysis of White et al's for the IVW and MR‐Egger approaches, using the methods described thus far. Full results are shown in Table 3.

**Table 3:** IVW and MR‐Egger regression analyses of the urate data.

Model Parameter	Est	S.E.	*t*‐value	*p*‐value	Heterogeneity statistics
IVW approach under model (14)					
$\beta_{IVW}^{ARE}$	0.222	0.093	2.43	0.021	*I* ^2^ = 53%, $\sigma_{\alpha }^{2}$ = 0.093
IVW approach under model (15)					
*β*_*IVW*_	0.163	0.067	2.45	0.020	*Q* = 63.9 (p=3 × 10^ − 4^), ${\hat{\varphi }}_{IVW}$= 2.13
IVW approach+SIMEX under model (15)					
*β*_*IVW*_	0.164	0.067	2.47	0.020	‐
MR‐Egger regression under model (20)					
*β*_0*E*_	0.008	0.005	1.61	0.11	‐
*β*_1*E*_	0.048	0.096	0.50	0.62	$Q^{\prime }$ = 58.6 (p=9 × 10^ − 4^), ${\hat{\varphi }}_{E}$= 2.02
MR‐Egger regression+SIMEX under model (20)					
*β*_0*E*_	0.008	0.005	1.59	0.12	‐
*β*_1*E*_	0.05	0.097	0.51	0.61	‐
Summary stats: $I_{GX}^{2}$ = 99%, $\bar{F}$ = 247, $Q-Q^{\prime }$ = 5.25, *Q*_*R*_ = 0.917					

**Figure 7:**
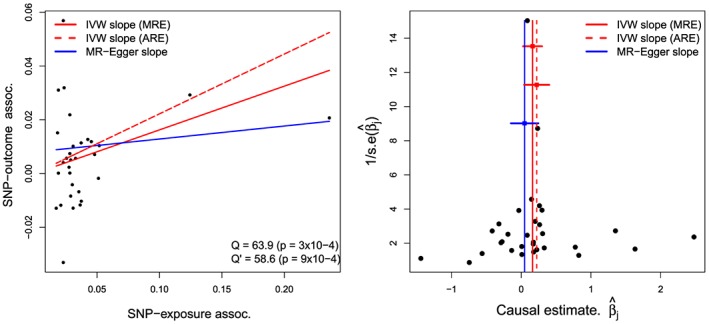
Left: scatter plot of the summary data estimates for the lipids data, with the IVW and MR‐Egger slope estimates. Right: corresponding funnel plot of the same data. ARE = additive random effects estimate ${\hat{\beta }}_{IVW}^{ARE}$, MRE = fixed effect/multiplicative random effects estimate ${\hat{\beta }}_{IVW}$.

Figure 7 (left) shows a scatter plot of the SNP‐exposure and SNP‐outcome association estimates, where the genetic data has been recoded in order to make the SNP‐exposure associations positive. The SNP‐outcome association estimates represent log‐odds ratios of CHD for a 1 standard deviation increase in plasma urate. Figure 7 (right) shows the corresponding funnel plot, which can be interpreted as discussed in Section 5. The vertical (and horizontal) lines show the causal effect estimates (and 95% confidence intervals) inferred by IVW and MR‐Egger regression.

We start our MR analysis by assuming that all variants are valid IVs and that the NOME, IV2, IV3 and InSIDE assumptions hold, which justifies the use of the fixed effect IVW model (10). The NOME assumption seems reasonable for these data under the IVW method, due a large mean *F* statistic across the genetic variants of 247 (with the weakest being 22 and the strongest being 4886). The IVW estimate ${\hat{\beta }}_{IVW}$ suggests that a one unit increase in plasma urate increases the log‐odds ratio of CHD by 0.163. However, there is evidence of heterogeneity among the 31 ratio estimates, Cochran's *Q* statistic on 30 degrees of freedom is 63.9, and the scale factor estimate ${\hat{\varphi }}_{IVW}$ = 2.13. We therefore relax assumption IV3 to allow each variant to have a pleiotropic effect (assuming it is ‘balanced’ under InSIDE) and perform random effects analysis. Applying the multiplicative random effects model (15) and so scaling up the variance of the fixed effect IVW estimate by ${\hat{\varphi }}_{IVW}$, the *p*‐value for ${\hat{\beta }}_{IVW}$ from a resulting t‐test is equal to 0.02. Applying the additive random effects model (14), we estimate the pleiotropic variance parameter $\sigma_{\alpha }^{2}$ to be 0.093, which yields an adjusted point estimate for ${\hat{\beta }}_{IVW}^{ARE}$ of 0.222 (an increase of 35%). Because both the fixed and additive random effects estimates should be targeting the same quantity under balanced pleiotropy plus InSIDE, this large difference suggests that the pleiotropy could have a significant directional component.

Given the large and significant *Q* statistic, we now apply MR‐Egger regression to these data to probe if there is indeed evidence for directional pleiotropy. We firstly assume that the pleiotropy is directional but constant and fit the fixed effect MR‐Egger model (16) under the NOME assumption. The NOME assumption seems reasonable for these data under the MR‐Egger method, because the $I_{GX}^{2}$ statistic is equal to 99.2% and so a less than 1% dilution in its causal estimate is expected.

**Figure 8:**
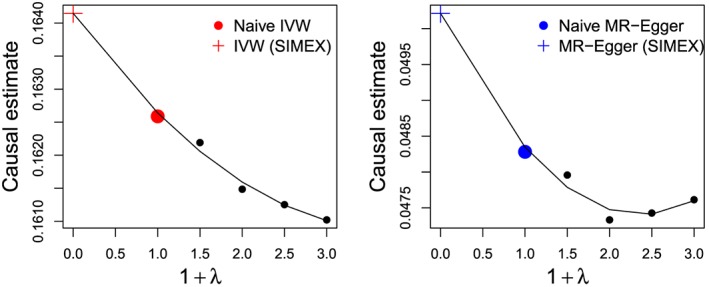
SIMEX adjustment applied to the IVW (left) and MR‐Egger (right) causal effect estimate. [Colour figure can be viewed at wileyonlinelibrary.com]

Under this model, MR‐Egger estimates a non‐zero mean pleiotropic effect of 0.008 and, because this is in the same direction, a reduced causal effect estimate, ${\hat{\beta }}_{1E}$, of 0.048. Calculating the residual heterogeneity about the MR‐Egger fit using Rücker's $Q^{\prime }$ statistic yields $Q^{\prime }=58.6$ with a p‐value of 9 × 10^ − 4^. We next allow for directional and heterogenous pleiotropy under the InSIDE assumption by fitting multiplicative random effects model (20), and scale up the variance of the fixed effect MR‐Egger estimates by a factor of ${\hat{\varphi }}_{E}=2.02$. Under this analysis, the p‐value for the mean pleiotropic effect (or intercept) parameter estimate ${\hat{\beta }}_{0E}$ is 0.12 and the p‐value for the causal effect estimate ${\hat{\beta }}_{1E}$ is 0.62. The ratio statistic *Q*_*R*_ is 0.917 indicating that the MR‐Egger model explains approximately 8% more of the variation in the SNP‐outcome association estimates than the IVW approach and the difference *Q*‐*Q′* is also large. Although the NOME assumption appears to be sensible for these data, for completeness, we use the SIMEX method to adjust for NOME violation in the IVW and MR‐Egger fits (under multiplicative random effects models only). R code to perform this method can be found in 17. The results are shown in Figure 8 and Table 3.

In summary, careful analysis of these data suggest that the large positive causal association of urate concentration with CHD risk is potentially unreliable due to the presence of directional pleiotropy manifesting itself as heterogeneity among the causal effect estimates. Whilst the application of an additive random effects model accounting for balanced pleiotropy (via the IVW estimate) increases the causal effect estimate further still, MR‐Egger regression suggests that the pleiotropy has a positive directional element, and consequently adjusts the causal estimate down towards zero. For these data, there is good evidence that MR‐Egger provides a better fit than IVW.

## 7 Discussion

In this paper, we have attempted to explain the many and varied assumptions that are necessary to apply standard meta‐regression and random effects methodology from mainstream meta‐analysis to summary data MR. We have also tried to explore the consequences for each method when some of the key assumptions (e.g. IV2, IV3, InSIDE and NOME) are violated, and clarified cases where the violation of one assumption (e.g. IV2 and VIS) acts to promote violation of another (e.g. InSIDE and NOME). We hope that those applying the IVW and MR‐Egger regression approaches can use this work to gain a more informed understanding of their strengths and limitations. One such limitation of MR‐Egger regression is that it is known to be far less efficient than the IVW method. For this reason, we also gave particular emphasis to examining the properties of the more established IVW estimate under both additive and multiplicative random effects models. We therefore view it as an important finding that the additive approach is less robust to directional pleiotropy, echoing the results of Henmi and Copas 15 for mainstream meta‐analyses affected by small study bias. This point was indeed highlighted in the analysis of the urate data affected in Section 6, whereby the IVW estimate actually increased under an additive random effects model in response to positive directional pleiotropy, rather than decreasing towards zero.

In practice, we would encourage users to report the full set of analyses described here, using the various heterogeneity and instrument strength statistics introduced as a guide to the relative importance that could be placed on each method for the data at hand.

### 7.1 Limitations

A major limitation of this work is that we have assumed throughout that the two‐sample assumptions hold, in particular that the SNP‐exposure and SNP‐outcome association estimates gleaned in separate populations provide information on a common (i.e. identical) set of parameters. This will not be true, for example, in the presence of gene‐environment interactions if the distribution of the environmental factor differs in the two samples. As further work, it is vital to understand the consequences for inference if this assumption is violated. In particular, it would be interesting to see whether the approaches discussed would remain valid under weaker assumptions. For example, if the parameters indexing the two population models were not identical, but were instead generated from a common distribution. A Bayesian implementation would then seem natural.

Another major limitation of this work is that we assumed additive linear models with no interaction terms for the SNP‐outcome association estimates. As shown by Didelez and Sheehan 33, such a model is required for consistent estimation of the causal effect. In practice (and in our real data example) MR analyses are generally performed with respect to a binary outcome yielding log‐odds ratios for the SNP‐outcome associations. In this case, when a causal effect between the exposure and outcome exists, ratio estimates of causal effect gleaned from individual variants will be attenuated towards the null by varying amounts due to the non‐collapsibility of the odds‐ratio 34. This is enough to invalidate our models in its own right. Furthermore, if the data were collected using case‐control sampling, as is also common, then log‐odds ratio estimates are also susceptible to ascertainment bias 35. It will be important to develop summary data methodology to properly account for these two issues as well.

A further limitation of our proposed framework for investigation of pleiotropy, is that it is purely based on statistical evidence, and ignores any *a priori* biological knowledge of possible pleiotropy for individual variants. As more is learned about the association between individual variants and multiple health outcomes, analyses that ignore this additional information will appear increasingly uninformed as well as inefficient.

Finally, we return to the InSIDE assumption. When explaining this assumption and exploring the impact of its violation on the IVW and MR‐Egger approaches, we assumed for simplicity that IV2 held. However, it is still possible in theory for InSIDE to hold even when IV2 is violated, albeit in fairly contrived circumstances. For example, suppose that IV2 and IV3 are violated for all variants but the pleiotropy via one route is perfectly negatively correlated with pleiotropy via the other, so that *α*_*j*_ =  − *ψ*_*j*_. Alternatively, suppose that IV3 is satisfied for all variants (*α*_*j*_= 0) but the magnitude of violation of IV2 is the same across variants (*ψ*_*j*_ = *ψ*). In both cases, the covariance between the instrument strength and direct effect is zero because the pleiotropy is constant, and Perfect InSIDE holds. Whether these facts could somehow be exploited to improve the performance of MR‐Egger regression is a subject for further investigation.

## Appendix 1

### 1.1

**Table A1:** Model parameters used for the theoretical calculations in Section 5.

Variant	*α*	*γ*	*ψ*
1	0.89	1.62	0.11
2	0.92	3.86	0.27
3	0.51	1.79	0.97
4	0.64	2.42	0.54
5	0.52	1.94	0.63
6	0.44	3.14	0.66
7	0.69	2.11	0.03
8	0.76	3.78	0.60
9	0.68	1.06	0.26
10	0.64	3.24	0.13
11	0.08	3.23	0.80
12	0.38	3.14	0.91
13	0.84	0.97	0.67
14	0.09	3.96	0.99
15	0.67	2.43	0.49
16	0.39	0.85	0.99
17	0.28	2.20	0.11
18	0.02	1.69	0.93
19	0.97	1.04	0.78
20	0.44	0.77	0.16
21	0.48	0.53	0.92
22	0.89	0.85	0.50
23	0.46	1.83	0.86
24	0.06	2.04	0.34
25	0.80	0.72	0.20
26	0.84	1.57	0.05
27	0.24	0.52	0.94
28	0.97	2.54	0.88
29	0.79	3.66	0.52
30	0.12	0.80	0.19
31	0.14	0.58	0.48
32	0.63	2.15	0.19
33	0.92	2.80	0.51
34	0.60	0.76	0.21
35	0.03	2.44	0.11
36	0.28	3.88	0.50
37	0.85	2.34	0.03
38	0.21	1.82	0.20
39	0.45	1.56	0.35
40	0.65	1.08	0.96
41	0.96	0.67	0.99
42	0.99	3.38	0.09
43	0.95	2.19	0.64
44	0.05	0.72	0.71
45	0.51	0.73	0.98
46	0.89	0.70	0.07
47	0.58	0.80	0.86
48	0.90	0.56	0.40
49	0.73	2.61	0.96
50	0.23	2.23	0.38
